# A principal components approach to parent-to-newborn body composition associations in South India

**DOI:** 10.1186/1471-2431-9-16

**Published:** 2009-02-24

**Authors:** Sargoor R Veena, Ghattu V Krishnaveni, Andrew K Wills, Jacqueline C Hill, Caroline HD Fall

**Affiliations:** 1 Epidemiology Research Unit, Holdsworth Memorial Hospital, Mysore, South India; 2 MRC Epidemiology Resource Centre, Southampton General Hospital, Southampton, UK; 3 Department of Obstetrics and Gynaecology, Cure International Hospital, Kabul, Afghanistan

## Abstract

**Background:**

Size at birth is influenced by environmental factors, like maternal nutrition and parity, and by genes. Birth weight is a composite measure, encompassing bone, fat and lean mass. These may have different determinants. The main purpose of this paper was to use anthropometry and principal components analysis (PCA) to describe maternal and newborn body composition, and associations between them, in an Indian population. We also compared maternal and paternal measurements (body mass index (BMI) and height) as predictors of newborn body composition.

**Methods:**

Weight, height, head and mid-arm circumferences, skinfold thicknesses and external pelvic diameters were measured at 30 ± 2 weeks gestation in 571 pregnant women attending the antenatal clinic of the Holdsworth Memorial Hospital, Mysore, India. Paternal height and weight were also measured. At birth, detailed neonatal anthropometry was performed. Unrotated and varimax rotated PCA was applied to the maternal and neonatal measurements.

**Results:**

Rotated PCA reduced maternal measurements to 4 independent components (fat, pelvis, height and muscle) and neonatal measurements to 3 components (trunk+head, fat, and leg length). An SD increase in maternal fat was associated with a 0.16 SD increase (β) in neonatal fat (p < 0.001, adjusted for gestation, maternal parity, newborn sex and socio-economic status). Maternal pelvis, height and (for male babies) muscle predicted neonatal trunk+head (β = 0. 09 SD; p = 0.017, β = 0.12 SD; p = 0.006 and β = 0.27 SD; p < 0.001). In the mother-baby and father-baby comparison, maternal BMI predicted neonatal fat (β = 0.20 SD; p < 0.001) and neonatal trunk+head (β = 0.15 SD; p = 0.001). Both maternal (β = 0.12 SD; p = 0.002) and paternal height (β = 0.09 SD; p = 0.030) predicted neonatal trunk+head but the associations became weak and statistically non-significant in multivariate analysis. Only paternal height predicted neonatal leg length (β = 0.15 SD; p = 0.003).

**Conclusion:**

Principal components analysis is a useful method to describe neonatal body composition and its determinants. Newborn adiposity is related to maternal nutritional status and parity, while newborn length is genetically determined. Further research is needed to understand mechanisms linking maternal pelvic size to fetal growth and the determinants and implications of the components (trunk v leg length) of fetal skeletal growth.

## Background

A large body of recent research has linked birth weight and simple body proportions at birth like ponderal index (weight/length^3^) to a range of diseases of adult life [1,2]. For example lower birthweight and ponderal index are associated with an increased risk of adult cardiovascular disease [3], and both low and high birthweight with an increased risk of type 2 diabetes mellitus and the metabolic syndrome [4-8]. These studies have led to intense interest in fetal growth and its determinants. Factors known to influence fetal growth include the 'maternal environment' (for example the mother's nutritional status and parity) and maternal and paternal genes [9,10].

Birthweight is a crude composite measure encompassing bone, fat, muscle and visceral mass. The proportions of these components may differ between populations [11], and have different determinants and associations with long-term outcomes. Relatively little is known in this area. Several studies have examined associations between the anthropometry of mothers (and sometimes fathers) and their babies [12-14], but such data can be difficult to interpret, due to strong correlations between the different indices of body composition. Others have overcome this by using principal components analysis [PCA], a statistical technique that transforms multiple observed variables into a smaller number of uncorrelated components that are interpretable [15-18]. In white Caucasian populations, PCA has fairly consistently identified fat (skinfold measurements) and skeletal size (length) as the main fetal components, with maternal body fat (skinfolds, BMI) and the skeletal size (height) of both parents respectively as their strongest predictors [15-17]. These findings have been corroborated by studies using more sophisticated measures of parental and newborn body composition, such as dual X-ray absorptiometry (DXA) [19-21].

An earlier study used PCA to describe neonatal anthropometry in relation to CVD risk factors in childhood an Indian population [18], but did not examine determinants of the neonatal components. Detailed anthropometry collected from mothers during pregnancy and their newborns in the Mysore Parthenon Study [22-24], enabled us to examine the body composition of mothers and babies in an Indian population. We included all body measurements recorded; for the mothers these included external pelvic diameters, which were of interest following an earlier Mysore study which showed that the risk of type 2 diabetes was increased in adult offspring of mothers with larger pelvic diameters [25], and for the babies the measurements included leg length in addition to crown-heel length, in view of recent interest in this measurement as a marker in adult life of poor growth in early development [26-29]. The main aim of this paper was to use anthropometry and principal components analysis (PCA) to describe maternal and newborn body composition, and associations between them. Our secondary aim was to compare associations of maternal and paternal height and BMI with neonatal body composition.

## Methods

### Study Sample

Between June 1997 and August 1998, 1539 women booking consecutively into the antenatal clinic at the Holdsworth Memorial Hospital (HMH), Mysore were screened [22]. They were eligible for the study if they were not known to be diabetic before pregnancy, planned to deliver at HMH, and had a singleton pregnancy of < 32 weeks gestation, determined by their last menstrual period (LMP) or a first trimester ultrasound scan. Of the women screened, 1233 women were eligible and 830 (67%) agreed to participate. The study was approved by the HMH research ethics committee and informed verbal consent was obtained from the women.

### Maternal measurements during pregnancy

At 30 ± 2 weeks gestation, detailed maternal anthropometric measurements including height, weight, head and mid-upper arm circumference (MUAC), skin fold thicknesses (triceps, biceps, subscapular and suprailiac) and external pelvic diameters (interspinous, intercristal and external conjugate) were measured by one of two trained observers using standardised methods. A Harpenden anthropometer (CMS instruments, London, UK) was used to measure external pelvic diameters. Measurements were taken in triplicate to the nearest 0.1 cm and average was used. The subject was asked to stand up straight with feet slightly apart and with the lower abdomen completely exposed to identify and mark the bony landmarks. Intercristal diameter was measured taking the widest transverse measurement, with the subject standing face on, by placing the tips of the callipers on the outer margins of the iliac crests. Interspinous diameter was measured, with the subject standing face on, by placing the tips of the callipers on the outer edges of the anterior superior iliac spines. External conjugate diameter was measured, with the subject standing side on, by placing one calliper tip on the anterior, upper margin of the pubic symphysis and the other on the spine of the last lumbar vertebra. Inter observer variation studies done at the time of data collection showed the amount of variation attributable to observers was only 0.004%, 0.8% and 1.2% for interspinous, intercristal and external conjugate measurements respectively. Maternal arm muscle area (AMA (cm^2^)) was calculated using the formula [(MUAC-π*triceps skinfold)^2^/4 π] [30]. We measured their husband's height and weight on the same day, or at the time of delivery, or at home. Maternal fasting plasma glucose concentration was measured as described previously [22]. Socio-economic status (SES) was assessed using Kuppuswamy score, a standard questionnaire method based on education, occupation and income of the main breadwinner [31]. We asked mothers about tobacco smoking, but none of the women were smokers.

### Deliveries and measurements of babies at birth

**Figure 1:**
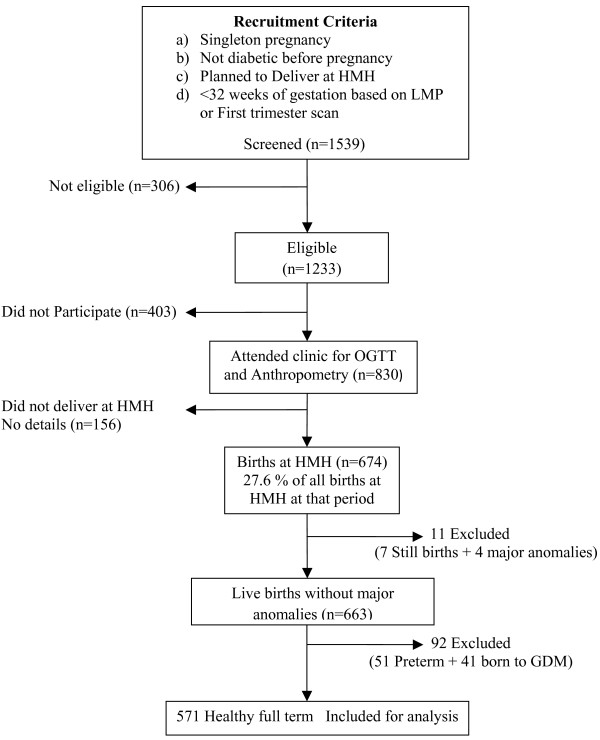
**Flow diagram depicting the study participants included for analysis**.

Six hundred and seventy four women went on to deliver their babies at HMH (81% of the participants), at a gestational age ranging from 29 to 44 weeks. Detailed newborn anthropometry was performed according to a standard protocol, within 72 hours of birth, by one of four trained observers. Weight was measured using a digital weighing scale (Seca, Germany) and crown-heel (CHL) and crown-rump (CRL) lengths using a Harpenden neonatal stadiometer (CMS instruments, London). Head, chest (xiphisternum), abdominal (umbilicus) and MUAC were measured with a blank tape, marked and measured against a fixed ruler. Skinfold thicknesses (triceps and sub-scapular) were measured using Harpenden skin-fold callipers (CMS instruments, London). Of 674 babies, 324 (48.1%) were male and 350 (51.9%) female. Seven still born babies, 4 with major congenital anomalies, 51 preterm babies (< 37 weeks gestation) and 41 babies born to mothers with gestational diabetes (GDM) were excluded from this analysis, leaving 571 mother-offspring pairs (Figure 1).

### Statistical Methods

Variables not satisfying normality assumptions were either log transformed (maternal weight, body mass index (BMI), maternal and neonatal skin-fold thicknesses) or square root transformed (supra-iliac skin-fold). Birth measurements (unadjusted for gestation) were used in PCA to describe neonatal body composition. Two PCAs were performed to describe the relationships between the neonatal variables and between the maternal variables. PCA is a dimension-reduction technique [32]. The aim was to reduce the anthropometric variables into smaller sets of independent factors that retained the information that distinguished individual differences between babies or mothers. The extracted components are a weighted linear composite of the original variables. The first component explains the most variance and each subsequent component attempts to capture the maximum of the remaining variance. Each component is orthogonal or independent of earlier components. The factor loadings reflect the contribution and direction of correlation between each variable and component. These are used for interpretation, and as such the retained factors should be biologically meaningful. Similar to a previous study [17], we examined the use of a factor rotation (varimax) to more clearly distinguish those variables that correlate highly with each component and aid interpretation. Standardised factor scores with a mean of 0 and standard deviation of 1 were ultimately produced using the rotated PCA. The correlation matrix was used to perform the PCA. Since similar factors were identified in both boys and girls, the sexes were pooled.

Factors were retained based on the % of variation explained (we investigated the plausibility of factors explaining < 10%), an inspection of the scree plots and also whether they were physiologically interpretable. The standardised neonatal and maternal factor scores were then used as variables to assess the mother-baby relationships. Associations between maternal components and each neonatal component were estimated using simple regression. The first set of models contained all maternal components together (they are independent so there is no confounding) and were also adjusted for gender. Effects in boys and girls were stratified where there was a statistically significant interaction (p < 0.05). Additional models were then produced with further adjustment for parity, SES and gestational age. In the regression analysis we have examined the associations between maternal components and neonatal leg length (standardised) instead of leg component as the correlation between neonatal leg factor and leg length and crown to heel is so strong that there ceases to be any extra additional information from using this factor as opposed to just leg alone, regardless of the correlation with crown to heel. And actual leg length is remarkably uncorrelated with factors 1 and 2 either.

Since we had limited anthropometric data on the father, comparisons between father-baby and mother-baby associations were restricted to parental height and BMI. Similar to the above, 2 sets of models were produced, the first adjusted only for gender (interactions were also tested), and in the second maternal and paternal variables included together in the model and further adjusted for parity, SES and gestational age. Stata v10 was used for all analyses.

## Results

Excluding preterm births from the analysis, anthropometric characteristics of the babies and mothers are summarised in Table 1. Mothers had a mean age of 24 years with a mean height of 154.7 cm and a median BMI of 23 kg/m^2^, and 51% were primiparous. Fathers had a mean age of 31 years with a mean height of 167.2 cm and a median BMI of 23 kg/m^2^.

**Table 1:** Characteristics of the study cohort

	**Mean**	**(SD)**
**Babies (n = 571)**		
		
Birthweight (kg)	2.877	(0.417)
Crown-heel length (cm)	48.7	(2.2)
Crown-rump length (cm)	32.0	(1.7)
Leg length (cm)	16.8	(1.5)
Head circumference (cm)	33.8	(1.3)
Mid upper arm circumference (cm)	10.3	(0.9)
Abdominal circumference (cm)	29.9	(1.9)
Chest circumference (cm)	31.9	(1.7)
Triceps skin-fold thickness (mm)*	4.1	(3.6, 4.7)
Subscapular skin-fold thickness (mm)*	4.3	(3.9, 4.9)
Gestational age (weeks)	39.5	(1.2)
		
**Mothers (n = 571)**		
		
Age (years)	23.6	(4.1)
Weight (kg)*	55.0	(49.5, 62.0)
Height (cm)	154.7	(5.4)
BMI (kg/m^2^)*	23.0	(20.9, 25.6)
Head circumference (cm)	53.4	(1.5)
Arm muscle area (cm^2^)	28.1	(4.8)
Triceps skin-fold thickness (mm)*	16.7	(12.1, 24.0)
Biceps skin-fold thickness (mm)*	8.7	(6.4, 12.6)
Subscapular skin-fold thickness (mm)*	24.4	(17.6, 33.2)
Suprailiac skin-fold thickness (mm)*	32.0	(23.2, 42.2)
Intercristal diameter (cm)	25.7	(2.5)
Interspinous diameter (cm)	23.5	(2.2)
External conjugate diameter (cm)	20.9	(2.4)
Parity (No (%))		
- 0	294	(51.5)
- 1	197	(34.5)
- 2 or more	80	(14.0)
Socio-economic status (score)	15.1	(5.0)
Fasting glucose (mmol/l)	4.5	(0.4)
		
**Fathers (n = 475)**		
		
Age (years)	31.3	(4.7)
Weight (kg)	64.5	(56.5, 71.0)
Height (cm)	167.2	(6.1)
BMI (kg/m^2^)	23.0	(20.3, 25.4)

*Transformed variable; values are median and inter quartile range

### Principal components analysis

#### Babies

PCA reduced the 10 birth measurements to three components (Table 2). The varimax rotation more clearly distinguished the newborn components. The first component (explaining 35% of variance) showed high correlations with birthweight, crown-heel and crown-rump length and head circumference, the second (33% of variance) showed high correlations with MUAC, triceps and subscapular skin folds and the third (15% of variance) showed a high correlation with leg length. We interpreted them as 'truncal skeleton+head', 'fat' and 'leg length' components respectively. Since actual leg length was so strongly correlated with neonatal factor 3 (r = 0.99), and also showed very little correlation with factors 1 (r = 0.01) and 2 (r = -0.00), we substituted it for the principal component in the regression analysis, to increase clinical relevance. Whether we used factor 3 or actual leg length made no difference to the overall interpretation.

**Table 2:** Principal components analysis: Unrotated and Varimax rotated components matrix of neonatal anthropometry.

	**Unrotated components**			**Varimax rotated components**		
**Neonatal anthropometry**	**UR1**	**UR2**	**UR3**	**R1**	**R2**	**R3**
Birthweight	**0.956**	0.029	-0.072	**0.735**	0.592	0.170
Crown-heel length	**0.745**	0.589	-0.148	**0.737**	0.164	0.596
Crown-rump length	**0.762**	-0.025	-0.542	**0.907**	0.202	-0.114
Leg length	0.195	**0.884**	0.416	0.014	0.003	**0.996**
Head circumference	**0.774**	0.036	-0.257	**0.732**	0.354	0.064
Abdominal circumference	**0.845**	-0.023	0.088	0.538	0.635	0.171
Chest circumference	**0.898**	0.050	-0.031	0.670	0.568	0.195
Mid-upper-arm-circumference	**0.829**	-0.213	0.109	0.479	**0.717**	0.009
Triceps skinfold	**0.746**	-0.364	0.355	0.226	**0.873**	-0.035
Subscapular skinfold	**0.739**	-0.303	0.418	0.189	**0.880**	0.044
Variance (%)	59.9	14.1	8.8	35.0	33.2	14.6
Cumulative Variance (%)	82.8			82.8		

Values are factor loadings (correlation co-efficient of the relationship between the factors produced and the individual anthropometric measurements); UR1, UR2 and UR3 represents the first, second and third unrotated factors extracted; R1, R2 and R3 represents the first, second and third rotated factors extracted. Values > 0.7 are highlighted. Variance is the percentage variance explained by each factor and cumulative variance is the percentage variance explained by all the three factors together.

#### Mothers

PCA reduced the 12 maternal anthropometric measurements to four components (Table 3), and again the rotated analysis produced clearer differentiation between measurements. The first component (explaining 39% of variance) was correlated with weight, BMI, and all four skin folds. The second (23% of variance) was correlated with all three pelvic measurements. The third and fourth components (12% and 11% of variance) were correlated with height and AMA respectively. We interpreted these components as maternal 'fat', 'pelvis', 'height' and 'muscle'.

**Table 3:** Principal component analysis: Unrotated and Varimax rotated components matrix of maternal anthropometry.

	**Unrotated components**				**Varimax rotated components**			
**Maternal anthropometry**	**M1**	**M2**	**M3**	**M4**	**RM1**	**RM2**	**RM3**	**RM4**
Weight	**0.921**	-0.003	0.253	0.064	**0.765**	0.345	0.337	0.315
Height	0.251	0.448	0.530	-0.540	-0.034	0.227	**0.882**	-0.066
Body mass index	**0.874**	-0.219	0.017	0.330	**0.842**	0.263	-0.062	0.372
Head circumference	0.441	0.119	0.643	-0.111	0.314	0.009	0.672	0.292
Arm muscle area	0.244	0.353	0.373	**0.772**	0.011	0.144	0.034	**0.948**
Triceps skinfold	**0.845**	-0.400	-0.071	-0.137	**0.919**	0.194	0.055	-0.113
Subscapular skinfold	**0.817**	-0.375	-0.076	-0.014	**0.881**	0.192	-0.011	-0.009
Biceps skinfold	**0.794**	-0.369	-0.012	-0.010	**0.862**	0.154	0.033	0.020
Suprailiac skinfold	**0.800**	-0.321	-0.003	-0.172	**0.839**	0.193	0.147	-0.097
Intercristal diameter	**0.775**	0.504	-0.239	-0.014	0.339	**0.870**	0.140	0.142
Interspinous diameter	0.610	0.656	-0.339	-0.042	0.113	**0.943**	0.095	0.086
External conjugate diameter	**0.717**	0.484	-0.328	-0.061	0.300	**0.872**	0.080	0.048
Variance (%)	50.6	15.3	9.9	8.9	39.0	23.4	11.7	10.6
Cumulative Variance (%)	84.7				84.7			

Values are factor loadings (correlation co-efficient of the relationship between the factors produced and the individual anthropometric measurements); M1, M2, M3, and M4 represents the first, second, third and fourth unrotated factors extracted; RM1, RM2, RM3, and RM4 represents the first, second, third and fourth rotated factors extracted. Values > 0.7 are highlighted. Variance is the percentage variance explained by each factor and cumulative variance is the percentage variance explained by all the four factors together.

### Relationships of maternal components to neonatal components

Of the four maternal components, fat was the strongest and only significant predictor of neonatal fat (Table 4). An SD increase in maternal fat was associated with a 0.16 SD increase in neonatal fat. Maternal pelvis and height, and for boys only, maternal muscle (p for sex interaction = 0.007), positively predicted neonatal truncal skeleton+head. None of the maternal components was related to neonatal leg length.

**Table 4:** Regression analysis of maternal components, fasting glucose, parity, socio-economic status and gestation as predictors of neonatal components

	Model 1*			Model 2**		
	**B**	**95% CI**	**p**	**B**	**95% CI**	**p**
**Neonatal fat (SD)**						
						
Maternal Fat (SD)	0.183	0.100, 0.267	< 0.001	0.157	0.073, 0.241	< 0.001
Maternal Pelvis (SD)	0.047	-0.035, 0.128	0.3	0.038	-0.043, 0.120	0.4
Maternal Height (SD)	0.006	-0.076, 0.088	0.9	-0.014	-0.101, 0.072	0.7
Maternal Muscle (SD)	0.059	-0.023, 0.142	0.2	0.034	-0.049, 0.119	0.4
Parity (0,1 and 2 or more)	0.155	0.041, 0.269	0.008	0.150	0.032, 0.268	0.013
Gestation (week)	0.057	-0.014, 0.128	0.1	0.060	-0.011, 0.130	0.09
Socio-economic status (Score)	0.017	0.0004, 0.033	0.045	0.019	0.001, 0.037	0.034
Fasting Glucose (mmol/l)	0.133	-0.065, 0.332	0.2	0.134	-0.074, 0.342	0.2
						
**Neonatal truncal skeleton and head (SD)**						
						
Maternal Fat (SD)	0.059	-0.022, 0.141	0.2	0.060	-0.020, 0.139	0.1
Maternal Pelvis (SD)	0.091	0.010, 0.172	0.029	0.094	0.017, 0.171	0.017
Maternal Height (SD)	0.147	0.065, 0.228	< 0.001	0.115	0.033, 0.197	0.006
Maternal Muscle (SD)						
Male	0.265	0.147, 0.383	< 0.001	0.267	0.154, 0.380	< 0.001
Female	0.093	-0.021, 0.206	0.1	0.051	-0.058, 0.161	0.4
Parity (0,1 and 2 or more)	-0.023	-0.136, 0.090	0.7	-0.016	-0.128, 0.095	0.8
Gestation (week)	0.277	0.211, 0.343	< 0.001	0.283	0.216, 0.349	< 0.001
Socio-economic status (Score)	0.018	0.002, 0.034	0.028	0.014	-0.002, 0.031	0.09
Fasting Glucose (mmol/l)	0.093	-0.102, 0.288	0.3	-0.005	-0.202, 0.192	0.9
						
**Neonatal Leg length (SD)**						
						
Maternal Fat (SD)	-0.042	-0.129, 0.044	0.3	-0.029	-0.117, 0.059	0.5
Maternal Pelvis (SD)	0.025	-0.062, 0.111	0.6	0.035	-0.051, 0.120	0.4
Maternal Height (SD)	0.061	-0.025, 0.148	0.2	0.065	-0.026, 0.156	0.2
Maternal Muscle (SD)	-0.074	-0.160, 0.013	0.1	-0.080	-0.169, 0.008	0.075
Parity (0,1 and 2 or more)	-0.068	-0.184, 0.047	0.2	-0.054	-0.178, 0.070	0.4
Gestation (week)	0.131	0.060, 0.201	< 0.001	0.143	0.069, 0.217	< 0.001
Socio-economic status (Score)	-0.006	-0.022, 0.011	0.5	-0.009	-0.027, 0.010	0.4
Fasting Glucose (mmol/l)	-0.002	-0.204, 0.199	0.9	-0.009	-0.230, 0.213	0.9

*Model 1: Adjusted for sex only. All four maternal components (which are uncorrelated) were included together, while parity, gestation socio-economic status and fasting glucose were examined singly. **Model 2: included sex and all the variables listed.

### Relationships of maternal parity, gestation, SES and glucose to neonatal components

Higher maternal parity was associated with increased neonatal fat (Table 4). Higher socio-economic status was associated with increased neonatal fat and truncal skeleton+head. Longer gestation was associated with larger neonatal truncal skeleton+head and longer leg length, but no increase in fat. There were no significant associations between maternal fasting glucose and neonatal components.

### Comparison of maternal and paternal associations with neonatal components

Maternal BMI predicted neonatal fat and truncal skeleton+head, more strongly than paternal BMI, and in the multivariable analysis, only maternal BMI remained a significant independent predictor of these neonatal components (Table 5). Paternal height predicted neonatal leg length more strongly than maternal height, and in the multivariable analysis remained the only significant predictor of neonatal leg length. Both maternal and paternal height predicted neonatal truncal skeleton+head, although both associations were of borderline statistical significance in the multivariate analysis.

**Table 5:** Regression analysis of maternal and paternal height and BMI as predictors of neonatal components

	Model 1**			Model 2***		
	**B**	**95% CI**	**p**	**B**	**95% CI**	**p**
**Neonatal fat (SD)**						
						
Maternal Height (SD)	-0.0002	-0.082, 0.082	0.9	0.027	-0.072, 0.126	0.6
Maternal BMI (SD)*	0.219	0.139, 0.299	< 0.001	0.203	0.108, 0.297	< 0.001
Paternal Height (SD)	-0.036	-0.128, 0.055	0.4	-0.046	-0.141, 0.049	0.3
Paternal BMI (SD)	0.116	0.025, 0.207	0.013	0.055	-0.039, 0.149	0.3
						
**Neonatal truncal skeleton and head (SD)**						
						
Maternal Height (SD)	0.124	0.044, 0.204	0.002	0.075	-0.014, 0.164	0.1
Maternal BMI (SD)*	0.178	0.099, 0.257	< 0.001	0.145	0.060, 0.230	0.001
Paternal Height (SD)	0.094	0.007, 0.181	0.030	0.081	-0.004, 0.166	0.063
Paternal BMI (SD)	0.095	0.009, 0.182	0.031	0.058	-0.027, 0.143	0.2
						
**Neonatal leg length (SD)**						
						
Maternal Height (SD)	0.083	0.0007, 0.166	0.048	0.064	-0.037, 0.165	0.2
Maternal BMI (SD)*	-0.055	-0.139, 0.028	0.2	-0.032	-0.129, 0.065	0.5
Paternal Height (SD)	0.147	0.055, 0.238	0.002	0.145	0.048, 0.241	0.003
Paternal BMI (SD)	-0.039	-0.131, 0.054	0.4	-0.014	-0.109 0.082	0.8

* Logged variable; **Model 1: Adjusted for sex only. Each variable shown was included singly in regression model; ***Model 2: All maternal and paternal variables were included together in the model, which were also adjusted for parity, gestation, sex and socio-economic status.

## Discussion

### Summary of main findings

**Figure 2:**
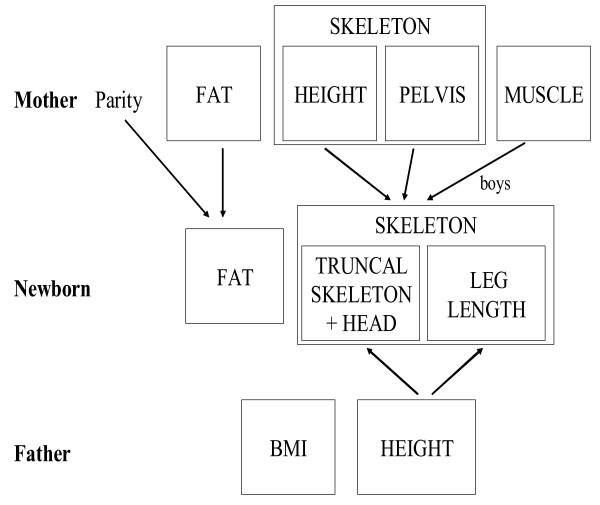
**Relationships between parental and newborn size**.

We used principal components analysis to study body composition in a large sample of healthy urban South Indian pregnant mothers and their full term newborn babies. Rotated PCA extracted four maternal components, which we interpreted as fat, pelvic size, height and muscle, and three neonatal (baby) components, interpreted as truncal skeleton+head, fat and leg length. We found that maternal fat predicted neonatal fat. The mother's pelvic size and height, and for boys maternal muscle, predicted neonatal truncal skeleton+head. In a comparison between maternal and paternal associations with newborn size, maternal (but not paternal) BMI predicted neonatal fat and neonatal truncal skeleton+head, while paternal (but not maternal) height predicted neonatal leg length. These findings are summarised in Figure 2.

### Strengths and weaknesses of the study

Strengths of the study were the detailed standardised anthropometric measurements for mothers and babies, including measurements not available in other studies (pelvic size for mothers and leg length for babies). Weaknesses were that there was no information on maternal weight gain during pregnancy, that the maternal measurements were collected during the third trimester of pregnancy (which means that maternal weight included fetal weight and that skinfolds could reflect fluid retention as well as adiposity), and that paternal measurements were limited to weight and height.

### Principal components analysis (newborns)

Several other studies have used PCA to examine newborn body composition in different populations [15-18]. Each used slightly different newborn measurements, although there was considerable overlap, and all had birth weight, crown-heel length, head circumference, mid-arm circumference and triceps and subscapular skinfolds. One of these was from another Indian population [18]. They all used a similar analysis to ours, putting all newborn measurements into the analysis together, and one study used the varimax rotation [17]. The studies using unrotated analyses [15,16,18] produced similar results, identifying two main components: firstly, overall size (with high loadings on all measurements) and secondly, a contrast between skeleton and fat (strong loadings in opposite directions for length and/or head versus skinfolds). The study that used varimax rotation, of white Caucasian babies from Exeter, UK [17] showed, as in our study, that this technique produced clearer differentiation between components. It too identified fat and skeleton (length+head), as the two main components. Our study differed from the Exeter study in differentiating leg length from crown-rump (trunk) length. Unlike knee-heel length in Exeter (the nearest equivalent in the Exeter study to leg length in our study) leg length did not have a strong factor loading in our neonatal R1 and was the only measurement with a strong factor loading in our neonatal R3 (Table 2). None of the other neonatal PCA studies had a separate measure of leg length.

### Principal components analysis (mothers)

The Exeter study also performed PCA on maternal measurements [17]. As in Mysore, the first two principal components loaded strongly on skeletal measurements (height and head circumference) and fat (skinfolds). In the Mysore data, we identified maternal pelvic size (all three pelvic measurements) and maternal muscle (arm muscle area) as two additional components. These data were not available in the Exeter study.

### Relationships between maternal and newborn body composition

We found that the maternal fat component was a strong, and the only, predictor of neonatal fat. This is consistent with other studies, that have used skinfolds, or other specific measurements of neonatal adiposity (including TOBEC and DXA) [19,20]. Along with our finding that maternal BMI, and not paternal BMI, predicted neonatal fat, this adds to other evidence that newborn adiposity is determined principally by factors operating through the mother [9,17]. These are likely to include the availability of nutrients, and their transfer to the fetus. As shown in other studies [9,17,33,34], neonatal fat was also increased in mothers of higher parity, which may reflect more efficient materno-fetal nutrient transfer in 'experienced' mothers. Women of higher parity tend to be heavier and fatter, which could explain some of this effect, but the association between parity and newborn fat remained statistically significant even in the multivariate analysis, which included all maternal body composition components, suggesting that parity has an independent effect on newborn adiposity. It is well known that babies born to diabetic mothers have increased body fat at birth [9] due to transfer of glucose across the placenta. The Exeter study, and the large, recently published multi-centre HAPO study, showed that even in non-diabetic pregnancies, neonatal adiposity is positively related to maternal fasting glucose concentrations [17,35]. We did not find this association in our data; although we have shown previously in this cohort that fasting glucose concentrations in non-diabetic mothers predicted newborn ponderal index [22]. The association between maternal fasting glucose, and the newborn fat component, was positive in our study but non-significant. This may be because the sample size was too small; we calculate that our study had only 50% power to detect an effect of the magnitude shown in the HAPO study.

The strongest maternal predictor of the neonatal truncal skeleton+head component was maternal height. Other maternal predictors, independent of this association, were pelvic size and (in male babies) muscle. The babies' truncal skeleton+head component was also positively related to paternal height and to gestation. The correlation with both maternal and paternal height supports other evidence that genetic factors make an important contribution to fetal skeletal growth [10,21,36,37].

The association of neonatal trunk length with maternal pelvic size, independent of other maternal measurements, is a new finding. External pelvic diameters are thought to be chiefly measurements of the bony skeleton, although they also incorporate subcutaneous fat. Our data suggest that pelvic size varies independently of maternal height, and of our index of maternal fat. Several studies have shown associations between maternal pelvic measurements and adult disease in the offspring, including stroke, hypertension, diabetes and cancer [25,38-41]. A recent study showed that the mother's intercristal diameter, but not height, predicted breast cancer in the adult female offspring [40]. The authors suggested that the growth of pelvic width is influenced by circulating oestrogen concentrations during puberty, whereas overall height is more strongly influenced by growth hormone. We are not able to comment on the implications of this for fetal development other than to say that the mother's pelvic size appears to reflect something more than her overall skeletal size and adiposity. More research into female pelvic development, and its relationship to fetal growth, is needed.

Relationships of maternal muscle to neonatal size have not been widely studied. Similar to our findings, maternal arm muscle area predicted neonatal length and not neonatal fat in a Peruvian population [42]. A recent study examining geographical variation in relationships between maternal body size and neonatal phenotype reported a significant association between maternal muscle and neonatal length in an African (DR Congo) population but not in other populations (including one other Indian population) [13]. Arm muscle area is an unsatisfactory measurement of maternal muscle mass, however, and more research is required to address associations between maternal muscle and fetal growth.

The third neonatal component, leg length, was predicted by paternal height. While the determinants of leg length at birth have been little studied, short leg length in childhood has been associated with maternal smoking and with poor childhood SES [43,44]. It has been described as a more sensitive indicator of the childhood environment than overall height [43,44]. Studies have also reported stronger associations of short leg length than of short overall height with elevated adult insulin resistance and cortisol concentrations and with an increased risk of diabetes and cardiovascular disease, suggesting important biological differences between these two length measurements [26-29]. The association between leg length and paternal height suggests that newborn leg length is at least partly genetically determined.

An exact equivalent of leg length was not available in the Exeter study, but leg length was measured using the same technique as ours in the Southampton Women's Survey [45]. Leg length in Southampton babies (mean = 16.5 cm) was almost identical to Mysore babies (16.7 cm) whereas crown-rump length was longer (mean = 33.6 cm in Southampton and 32.0 cm in Mysore), (Sarah Crozier, personal communication). One possible explanation for our findings is that the greater muscularity of UK babies (including larger buttock muscularity) makes leg length (as measured by crown-heel length minus crown-rump length) appear falsely short in the UK babies. However, a fetal ultrasound study in India showed comparable femur length measurements to western studies, even though birthweight was lower [46]. In another Indian study, crown-heel length was relatively preserved (-1.01 SD compared with UK babies), even though birthweight was considerably lighter (-1.74 SD) [11]. Another interpretation of our data is therefore that leg growth is preserved in Indian babies, even in the presence of marked growth restriction. The issue of whether the small Indian baby is growth-restricted or appropriate for maternal size is a much-debated question. Birth weight has risen in the upper socio-economic strata of the Indian population, as maternal heights have increased, suggesting that the small babies of small mothers have not realised their 'potential'. Lower birth weight is associated with higher infant mortality in India, and with higher cardiovascular disease risk factors in Indian children [47]. There are also strong associations between low birthweight and childhood stunting, lower educational achievement, and lower birthweight in the next generation, independent of early-life socio-economic status [48]. Hence, we think that growth restriction is an appropriate description.

## Conclusion

In conclusion, the neonatal anthropometric variables in Mysore showed a broadly similar underlying variance structure to that shown in other studies. Neonatal leg length was an important independent component in the babies, and pelvic diameters formed an independent component in the mothers; neither of these measurements were included in earlier PCA studies. Our findings are in keeping with previous research suggesting that newborn adiposity is influenced by the mother's nutritional status and parity, while neonatal length is genetically influenced. Our study has indicated areas for future research, including pelvic growth and the mechanisms by which maternal pelvic size are related to fetal growth, and the implications of truncal skeletal growth versus leg growth in the fetus. The neonatal components we identified will be useful in studying associations between other maternal exposures (such as micronutrient status) and fetal development, and between newborn size and later outcomes such as cognitive function and risk markers for adult disease.

## Competing interests

The authors declare that they have no competing interests.

## Authors' contributions

SRV conceived the idea of performing a principal components analysis, contributed to the analysis and interpretation of the data and drafted the manuscript. GVK recruited the mothers, carried out maternal and newborn measurements and assisted in writing the manuscript. AKW carried out the statistical analysis, and assisted in data interpretation and drafting of the manuscript. JCH recruited the mothers, carried out maternal and newborn measurements, coordinated the pregnancy phase of the study and also assisted in the writing of the manuscript. CHDF designed the cohort study, contributed to the data interpretation and assisted in drafting the manuscript. All authors have read and approved the final manuscript.

## Pre-publication history

The pre-publication history for this paper can be accessed here:


